# Supplementary material for: Characterization of RAGE and CK2 Expressions in Human Fetal Membranes
Source: Int J Mol Sci. 2023 Feb 17;24(4):4074. doi: 10.3390/ijms24044074 (PMC9966553; doi:10.3390/ijms24044074)
Supplement: Supplementary file 1 [file ijms-24-04074-s001.zip › Figure S3.pdf]

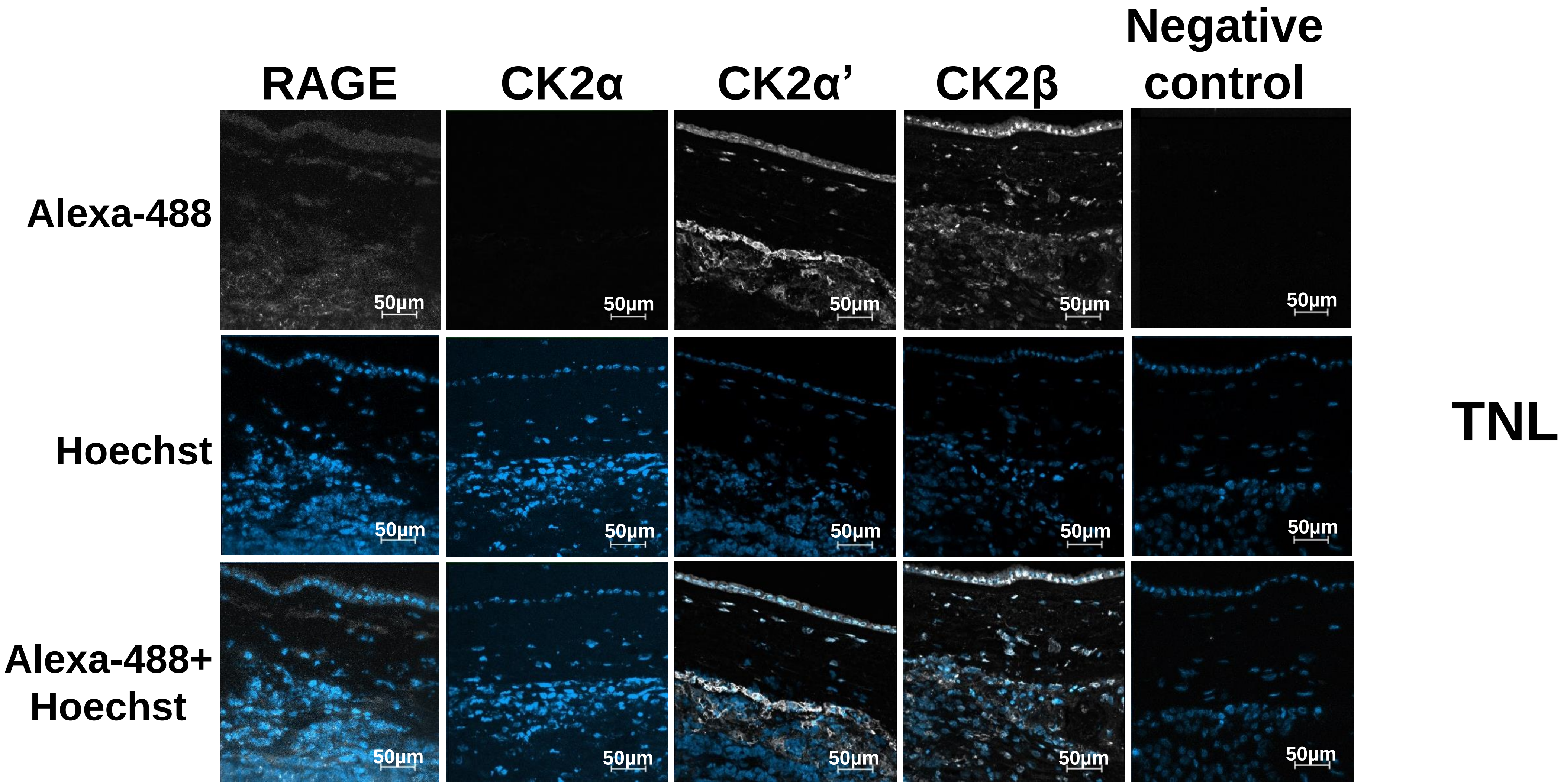

Figure S3. RAGE and CK2α, CK2α′, and CK2β localizations in human fetal membranes at term with no labor (TNL) by immunofluorescence.
